# The Thioredoxin-Regulated α-Amylase 3 of *Arabidopsis thaliana* Is a Target of S-Glutathionylation

**DOI:** 10.3389/fpls.2019.00993

**Published:** 2019-07-31

**Authors:** Libero Gurrieri, Luca Distefano, Claudia Pirone, Daniel Horrer, David Seung, Mirko Zaffagnini, Nicolas Rouhier, Paolo Trost, Diana Santelia, Francesca Sparla

**Affiliations:** ^1^Department of Pharmacy and Biotechnology FaBiT, University of Bologna, Bologna, Italy; ^2^Department of Plant and Microbial Biology, University of Zürich, Zurich, Switzerland; ^3^John Innes Centre, Norwich, United Kingdom; ^4^Université de Lorraine, Inra, IAM, Nancy, France

**Keywords:** α-amylase 3, post-translational redox modifications, S-glutathionylation, disulfide, cysteine p*K*a, glutaredoxin, thioredoxin

## Abstract

Reactive oxygen species (ROS) are produced in cells as normal cellular metabolic by-products. ROS concentration is normally low, but it increases under stress conditions. To stand ROS exposure, organisms evolved series of responsive mechanisms. One such mechanism is protein S-glutathionylation. S-glutathionylation is a post-translational modification typically occurring in response to oxidative stress, in which a glutathione reacts with cysteinyl residues, protecting them from overoxidation. α-Amylases are glucan hydrolases that cleave α-1,4-glucosidic bonds in starch. The Arabidopsis genome contains three genes encoding α-amylases. The sole chloroplastic member, *At*AMY3, is involved in osmotic stress response and stomatal opening and is redox-regulated by thioredoxins. Here we show that *At*AMY3 activity was sensitive to ROS, such as H_2_O_2_. Treatments with H_2_O_2_ inhibited enzyme activity and part of the inhibition was irreversible. However, in the presence of glutathione this irreversible inhibition was prevented through S-glutathionylation. The activity of oxidized *At*AMY3 was completely restored by simultaneous reduction by both glutaredoxin (specific for the removal of glutathione-mixed disulfide) and thioredoxin (specific for the reduction of protein disulfide), supporting a possible liaison between both redox modifications. By comparing free cysteine residues between reduced and GSSG-treated *At*AMY3 and performing oxidation experiments of Cys-to-Ser variants of *At*AMY3 using biotin-conjugated GSSG, we could demonstrate that at least three distinct cysteinyl residues can be oxidized/glutathionylated, among those the two previously identified catalytic cysteines, Cys499 and Cys587. Measuring the p*K*_a_ values of the catalytic cysteines by alkylation at different pHs and enzyme activity measurement (p*K*_a1_ = 5.70 ± 0.28; p*K*_a2_ = 7.83 ± 0.12) showed the tendency of one of the two catalytic cysteines to deprotonation, even at physiological pHs, supporting its propensity to undergo redox post-translational modifications. Taking into account previous and present findings, a functional model for redox regulation of *At*AMY3 is proposed.

## Introduction

Starch is the most abundant non-structural carbohydrate in plants. Mostly depending on its lifetime, starch can be distinguished in storage and transitory starch. Transitory starch is typically produced in photosynthetic organs where it is accumulated during the day and degraded in the following dark period. Transitory starch metabolism has been deeply studied over the last decades and its biosynthetic and degradation pathways have been detailed (Zeeman et al., 2010; Stitt and Zeeman, 2012; Skryhan et al., 2018). Several enzymes emerged as fundamental for leaf starch turnover and when their corresponding genes are knocked out, plants show starch-less or starch-excess phenotypes (Yu et al., 2001; Kötting et al., 2005, 2009; Fulton et al., 2008; Streb et al., 2009; Crumpton-Taylor et al., 2013). Another set of enzymes, the lack of which does not lead to starch-related phenotypes in leaves under standard growth conditions, was found localized in chloroplasts of guard cells (Outlaw, 2003; Horrer et al., 2016) as well as involved in leaf starch degradation pathway occurring in response to abiotic stresses (Zeeman et al., 2004; Valerio et al., 2011; Monroe et al., 2014; Zanella et al., 2016; Thalmann et al., 2016; Horrer et al., 2016).

Among the three α-amylases encoded by the Arabidopsis genome, *At*AMY3 is not required for normal starch breakdown in mesophyll cells and its knock-out mutant does not present a starch-excess phenotype (Yu et al., 2005). In contrast to the other α-amylases, *At*AMY3 localizes in chloroplast’s stroma of both mesophyll and guard cells (Yu et al., 2005; Delatte et al., 2006) and is regulated by thioredoxins (TRXs) through the formation of a disulfide bridge between Cys499 and Cys587 with a midpoint redox potential at pH 7.9 of −329 mV (Seung et al., 2013).

TRXs are small oxidoreductases found in various subcellular compartments. In chloroplasts, TRXs are reduced by electrons provided by photosystem I (PSI) and relayed by Ferredoxin (FDX), FDX:TRX reductase (FTR) and TRX. Through this redox chain, TRX-target enzymes achieve their modulation by light (Michelet et al., 2013; Balsera et al., 2014). The activation of *At*AMY3 by TRX-*f*1 is therefore consistent with its role in starch degradation at day in response to stress (Seung et al., 2013) as well as its presence in guard cells, where starch metabolism follows an opposite rhythm in comparison to mesophyll cells, mobilizing starch in the light to produce malate and/or sucrose, which contribute to increase guard cell osmolarity, turgor and stomatal opening (Vavasseur and Raghavendra, 2005; Lawson et al., 2014; Horrer et al., 2016; Santelia and Lawson, 2016; Santelia and Lunn, 2017).

In addition to a TRX-mediated redox regulation, thiol groups of cysteine residues can be modified by other redox modifications such as S-glutathionylation and S-nitrosylation (for a comprehensive review see Zaffagnini et al., 2019). These redox post-translational modifications (PTMs) are favored by the propensity of a cysteine residue to exist in a deprotonated form (thiolate anion; -S^–^) even at physiological pH values. Consequently, reactive cysteines are often characterized by p*K*_a_ values lower than the p*K*_a_ value of non-reactive cysteine thiol (i.e., below ≃7.0 and close to ≃8.5, respectively).

In chloroplasts, environmental changes such as drought, exposure to intense light or high temperature, are rapidly perceived and the production of reactive oxygen species (ROS) increases rapidly (Cruz de Carvalho, 2008; Chaves et al., 2009; Farooq et al., 2009; Sharma et al., 2012; Suzuki et al., 2012; Noctor et al., 2018). Compared to singlet oxygen and superoxide radical, hydrogen peroxide (H_2_O_2_) has received particular attention, being a long-lived molecule (Mittler and Zilinskas, 1991) that can possibly diffuse over quite long distances acting as signal molecule (Bienert et al., 2007; Sharma et al., 2012). However, at high concentrations, H_2_O_2_ can act as damaging molecule, rapidly oxidizing thiolate anions and leading to the formation of sulfenylated cysteines (-SOH). Under this first oxidative state, sulfenylated cysteines can be further oxidized to sulfinic (−SO_2_H) and sulfonic (−SO_3_H) acids (Roos and Messens, 2011; Roos et al., 2013; Trost et al., 2017), both considered irreversible oxidation states leading to protein degradation (Poole, 2015; Zaffagnini et al., 2019). Sulfenic acid can react with a thiol group of a protein or of low molecular weight molecules as reduced glutathione (GSH) leading to protein disulfide or protein S-glutathionylation, respectively (Ito et al., 2003; Dixon et al., 2005; Michelet et al., 2005; Zaffagnini et al., 2012a).

*At*AMY3 was found sulfenylated upon H_2_O_2_ treatment *in vivo* experiments (De Smet et al., 2018), and recombinantly expressed AMY1 of barley was found glutathionylated at Cys95 (Søgaard et al., 1993; Juge et al., 1996). Cys95 of barley AMY1 corresponds to Cys587 in *At*AMY3, a residue required for the optimal catalytic rate of the Arabidopsis enzyme, and involved in the formation of the thioredoxin regulated disulfide bridge together with Cys499 (Seung et al., 2013).

Reduced glutathione is one of the redox molecules that, together with ascorbate and a set of ROS-scavenging enzymes, contributes to cellular redox homeostasis (Shigeoka et al., 2002; Mittler et al., 2004; Foyer and Shigeoka, 2011; Sharma et al., 2012; Noctor and Foyer, 2016; Foyer and Noctor, 2016). GSH plays an important role against ROS-induced oxidative damages, as it is able to scavenge H_2_O_2_ leading to the formation of oxidized glutathione (GSSG). The balance between GSH and GSSG is central for cellular redox state, mirroring the redox state of a cell that can change in response to internal and external stimuli (Foyer and Noctor, 2016). In response to stress, the raise of H_2_O_2_ concentration causes accumulation of GSSG due to GSH-scavenging effect. The GSSG concentrations may become high enough to allow protein glutathionylation, helping to prevent cysteine overoxidation by other oxidants (Michelet et al., 2005; Noguera-Mazon et al., 2006; Tarrago et al., 2009).

In analogy with TRXs, which reduce regulatory disulfide bridges, glutaredoxins (GRXs) remove glutathione from modified enzymes (Rouhier et al., 2008; Zaffagnini et al., 2012b). GRXs are oxidized by the protein substrates, the activity of which is usually restored upon reduction. The glutathionylated GRX is reduced using another GSH molecule forming GSSG.

In this study, we have expressed the recombinant *At*AMY3 and conducted a biochemical characterization of its sensitivity to oxidizing and reducing treatments in the presence of glutathione and H_2_O_2_. We propose a fine-tuned regulatory mechanism for *At*AMY3 activity involving both S-glutathionylation and disulfide bridge formation.

## Materials and Methods

### *In silico* Analysis of α-Amylases

The amino acid sequences of *Arabidopsis thaliana* α-amylase 3 (UniProtKB: Q94A41; *At*AMY3); *A. thaliana* α-amylase 1 (UniProtKB: Q8VZ56; *At*AMY1); *A. thaliana* α-amylase 2 (UniProtKB: Q8LFG1; *At*AMY2) and *Hordeum vulgare* α-amylase type A isozyme (UniProtKB: P00693; *Hv*AMYA) were aligned with Espript^1^ (Robert and Gouet, 2014). Sequence identities among considered α-amylases calculated by Clustal Omega (Sievers et al., 2011) were the following: 46% for *At*AMY3 vs. *At*AMY1; 49% for *At*AMY3 vs. *At*AMY2; 47% for *At*AMY3 vs. *Hv*AMYA.

### Expression and Purification of Recombinant Proteins

Wild-type and mutated forms of *At*AMY3 were expressed and purified as described in Seung et al. (2013). Protein purity was assessed by 12.5% SDS-PAGE. Pure recombinant proteins were quantified by their absorbance at 280 nm (Nanodrop; Thermo Fisher Scientific) using molar extinction coefficients and molecular masses calculated from the amino acid sequences of the recombinant enzymes with the online ProtParam tool (Gasteiger et al., 2005). The recombinant *A. thaliana* GRX C5 and poplar GRX S12 were expressed in *Escherichia coli* and purified as described in Couturier et al. (2009, 2011).

### Enzyme Activity Assays

The enzymatic activity of *At*AMY3 was measured using the α-Amylase Assay Kit (Ceralpha Method) from Megazyme (Megazyme, Ireland) according to the manufacturer’s instructions. Briefly, a mixture composed by an equal volume of *At*AMY3 in 100 mM Tricine-NaOH, pH 7.9 and the artificial substrate blocked *p*-nitrophenyl maltoheptaoside (B-PNPG7) plus α-glucosidase was incubated at 40°C. After incubation, the reaction was blocked by adding 20-volume of Stopping Reagent (1% Tris, pH 11.0). The absorbance of the samples was evaluated at 400 nm using a spectrophotometer and subtracting the absorbance of blank sample treated under the same condition but without *At*AMY3. An extinction coefficient at 400 nm for *p*-nitrophenol of 18.1 mM^–1^ cm^–1^ was used.

### Oxidative Treatments

All oxidative treatments were performed on freshly reduced *At*AMY3. Following 90 min of incubation at 37°C in the presence of 40 mM dithiothreitol (DTT), protein sample was desalted using NAP-5 column (GE Healthcare) in 100 mM Tricine-NaOH, pH 7.9 and quantified by absorbance at 280 nm using an extinction coefficient of 185420 M^–1^ cm^–1^ and molecular weight of 96976.53 Da (Nanodrop; Thermo Fisher Scientific). Sample was brought to the desired concentration (≈20 μM) either by dilution or by concentration through Amicon-Ultra device (Millipore; cut-off 10 kDa).

Inactivation kinetics by H_2_O_2_ of *At*AMY3 were performed by incubating the reduced recombinant enzyme at 25°C in 100 mM Tricine-NaOH, pH 7.9, in the absence (control) or presence (treated sample) of 0.1 mM, 0.25 mM, 0.5 mM and 1 mM H_2_O_2_. At different time points, the enzyme activity was measured on 1:5 diluted samples using the α-Amylase Assay Kit (Ceralpha Method; Megazyme, Ireland). The data are expressed as percentage of residual activity relatively to the initial activities (*t* = 0) measured on the control samples.

Treatments with H_2_O_2_, H_2_O_2_ plus GSH and GSSG were performed by incubating reduced *At*AMY3 at 25°C in the presence of 0.1 mM, 0.5 mM or 1 mM H_2_O_2_ for 1 h; 1 mM H_2_O_2_ and 5 mM GSH for 1 h; 50 μM, 0.25 mM, 1 mM or 5 mM GSSG for 30 min. After the treatments, the enzyme activity was measured on 1:5 diluted samples using the α-Amylase Assay Kit (Ceralpha Method; Megazyme, Ireland). The reversibility of the oxidative treatments was tested by incubating the samples with 80 mM DTT for an additional 30 min at 25°C. After the treatments, the enzyme activity was measured on 1:5 diluted samples using the α-Amylase Assay Kit (Ceralpha Method; Megazyme, Ireland).

The data are reported as percentage of activity relative to control samples incubated without oxidants under the same condition.

### Reactivation of Glutathionylated *At*AMY3 by GSH, GRXs, and TRX

Freshly reduced *At*AMY3, obtained as previously described, was incubated with 5 mM GSSG for 90 min at 25°C. After GSSG incubation, sample was desalted through NAP-5 column (GE Healthcare) in 100 mM Tricine-NaOH, pH 7.9 and brought to the desired concentration (20 μM) through Amicon-Ultra device (Millipore; cut-off 10 kDa). Sample concentration was determined by absorbance at 280 nm (Nanodrop; Thermo Fisher Scientific).

Reactivation treatments of glutathionylated *At*AMY3 were performed by incubating samples for 30 min at 25°C in the presence of 50 μM, 0.2 mM, 1 mM, 2 mM, 5 mM, and 7 mM GSH. Samples were then diluted 5-fold and the activity assayed as described above.

Reactivation assays of GSSG-inhibited *At*AMY3 performed in the presence of poplar GRX S12 and *A. thaliana* GRX C5, and commercially available *E. coli* TRX (Sigma-Aldrich; protein id AAA24693), were conducted through 5, 15, and 30 min of incubation of 20 μM glutathionylated *At*AMY3 in the presence of 2 mM GSH with or without 5 μM GRXs, or in the presence of 0.2 mM DTT with or without 10 μM TRX. Upon incubation, enzyme activity was measured on 5-fold diluted samples as described above.

All data are expressed as percentages of activity relative to the fully reduced sample obtained by incubating glutathionylated *At*AMY3 in presence of 80 mM DTT for 30 min at 25°C.

### Quantification of Thiol Groups

Protein thiol quantification was performed on both reduced and GSSG-treated *At*AMY3. Freshly reduced *At*AMY3, obtained by 90 min incubation at 37°C in the presence of 40 mM DTT, was desalted in 100 mM Tricine-NaOH, pH 7.9 with NAP-5 column (GE Healthcare) and then incubated with 5 mM GSSG for additional 90 min at 25°C. After GSSG incubation sample was desalted in 100 mM Tricine-NaOH, pH 7.9 with NAP-5 column (GE Healthcare).

Reduced and GSSG-treated enzyme was incubated in the presence of 0.5 mM 5,5′-dithiobis (2-nitrobenzoic acid) (DTNB) for 1 h at room temperature. The number of free and solvent accessible thiol groups under the two tested conditions, was calculated from the molar ratio between the absorbance at 412 nm [molar extinction coefficient of 14150 M^–1^ cm^–1^ for 2-nitro-5-thiobenzoate(thiolate) dianion] and the absorbance at 280 nm (molar extinction coefficient of 185420 M^–1^ cm^–1^ for *At*AMY3) (Conway et al., 2004).

### Biotinylated GSSG Assay

Biotin-conjugated GSSG (BioGSSG) was freshly prepared by incubating for 1 h at room temperature 50 μl of 32 mM GSSG with 50 μl of 48 mM EZ-Link Sulfo-NHS-Biotin (Thermo Fisher Scientific) in 50 mM potassium phosphate buffer pH 7.2. The reaction was stopped by the addition of 35 μl of 0.6 M NH_4_HCO_3_.

Wild-type *At*AMY3 and all single cysteine to serine mutants were desalted in 100 mM Tricine-NaOH, pH 7.9 by NAP-5 columns (GE Healthcare). Protein concentrations were determined by absorbance at 280 nm (Nanodrop; Thermo Fisher Scientific) and 2 μM enzymes were incubated at room temperature in the presence of 2 mM BioGSSG at 25°C. After 1 h incubation, each sample was divided into two aliquots. One aliquot was treated with 80 mM DTT for 30 min to assess the reversibility of the reaction, whereas the second aliquot was transferred into a tube containing SDS-loading buffer 1X without reducing agent and in presence of 100 mM iodoacetamide (IAM) and 20 mM *N*-ethylmaleimide (NEM) to block cysteine reactivity. Negative control samples were incubated with the alkylating agents IAM (100 mM) and NEM (20 mM) for 30 min in the dark before incubation in the presence of 2 mM BioGSSG.

In the BioGSSG assays performed on pre-reduced or pre-oxidized enzymes, the recombinant proteins were incubated with 40 mM DTT or 40 mM *trans-*4,5-Dihydroxy-1,2-dithiane (DTTox) for 4 h at room temperature. Reduced and oxidized enzymes were desalted on a NAP-5 column (GE Healthcare) pre-equilibrated in 100 mM Tricine-NaOH, pH 7.9 before proceeding with BioGSSG analysis as described above.

Following incubations, protein samples were further divided into two aliquots and loaded on two denaturing non-reducing 12.5% SDS-PAGE. One gel was analyzed by Coomassie staining while the second gel was transferred to a nitrocellulose membrane and analyzed by Western blot using monoclonal anti-biotin antibodies (Sigma-Aldrich) diluted 1:3800. Peroxidase-conjugated secondary antibodies (Sigma-Aldrich) were diluted 1:2000 and were used for the detection by ECL Western Blotting Detection Reagent (GE Healthcare), following the manufacturer’s instruction.

### Determination of the p*K*_a_ of the Catalytic Cysteines

The p*K*_a_ values of the active-site cysteines were determined by measuring the pH dependence of the rate of reaction of *At*AMY3 as reported in Kallis and Holmgren (1980). Briefly, for each pH value (100 mM Sodium citrate for pH 4.0–5.5; 100 mM MES for pH 6.0–6.5; 100 mM Tris–HCl for pH 7.0–9.0; 100 mM Glycine for pH 10.0–12.0), 8 μM recombinant enzyme was incubated with or without a 10-fold excess of the alkylating reagent IAM with respect to the total thiol content of the protein sample. After 20 min incubation, samples were diluted 5-fold and α-amylase activity was measured with the artificial substrate BPNPG7 (Megazyme). *At*AMY3 did not undergo irreversible changes in the analyzed pH range, except for the pH values of 4.0, 11.0, and 12.0, which inhibited *At*AMY3 control samples by about 56% on average. The residual activity, expressed as a percentage of inhibition between IAM-treated and untreated samples, was plotted against pH.

Data sets were fitted by non-linear regression using the following equations with one-p*K*_a_ or two-p*K*_a_ dependence (Karala et al., 2010):

(a) one-p*K*_a_ dependence:

$$\%remainingactivity=100-\left(a\times \frac{10^{\left(\mathrm{pH}-p\,K\,a\right)}}{\left(1+10^{\left(\mathrm{pH}-p\,K\,a\right)}\right)}\right)$$

(b) two-p*K*_a_ dependence:

$$\%remainingactivity=100-\left(a\times \frac{10^{\left(\mathrm{pH}-p\,K\,a\,1\right)}}{\left(1+10^{\left(\mathrm{pH}-p\,K\,a\,1\right)}\right)}+b\times \frac{10^{\left(\mathrm{pH}-p\,K\,a\,2\right)}}{\left(1+10^{\left(\mathrm{pH}-p\,K\,a\,2\right)}\right)}\right)$$

## Results

### GSH Protects *At*AMY3 From Irreversible Inactivation Mediated by H_2_O_2_

**FIGURE 1:**
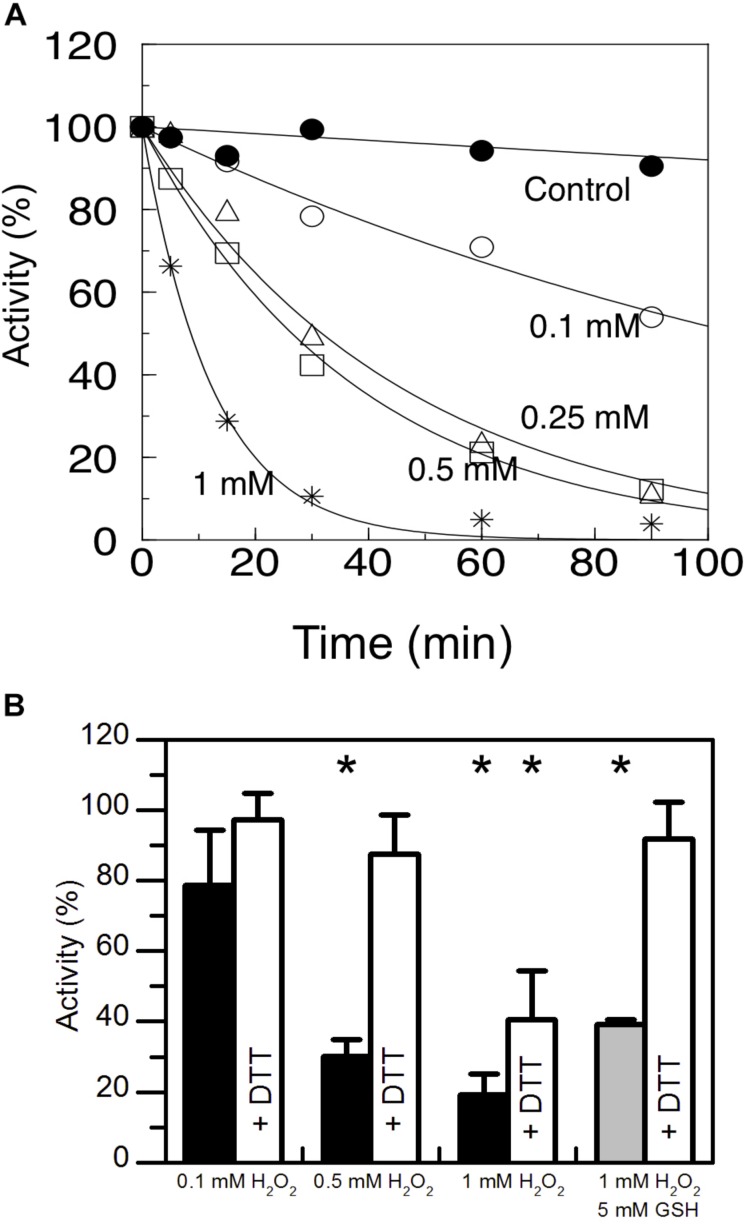
Sensitivity of *At*AMY3 to oxidative treatments. **(A)** Time- dependent inactivation of *At*AMY3 by 0.1 mM H_2_O_2_ (open circles), 0.25 mM H_2_O_2_ (open triangles), 0.5 mM H_2_O_2_ (open squares), 1 mM H_2_O_2_ (asterisks). Tricine-NaOH buffer was used as a control (closed circles). At the indicated time points, α-amylase activity was measured on aliquots of the incubation mixtures. Activities are expressed as percentages of the control activity measured at *t* = 0. Values are mean ± SD (*n* = 3; *SD* < 10% are omitted for clarity). **(B)** Inactivation of *At*AMY3 after 1 h incubation with 0.1 mM, 0.5 mM, 1 mM H_2_O_2_ (black bars), and 1 mM H_2_O_2_ plus 5 mM GSH (gray bar). The recovery of *At*AMY3 activity (white bars) was tested by incubating treated samples for additional 30 min with 80 mM DTT. Activities are expressed as percentages of control (untreated) sample. Data are reported as mean ± SD (*n* = 3). Significant reduction in enzyme activity compared to the control activity was determined based on *P*-values obtained from Student’s *t* test. ^*^P < 0.01.

*At*AMY3 has a well-documented function in the chloroplasts of mesophyll cells in response to osmotic stress, and in guard cells during stomatal opening (Yu et al., 2005; Horrer et al., 2016; Thalmann et al., 2016). Under osmotic stress conditions, ROS production can exceed the scavenging ability of plants (Chaves et al., 2009), becoming harmful molecules that lead to oxidative damage. For this reason, the susceptibility of *At*AMY3 to redox modulation was analyzed by measuring the enzymatic activity in the presence of increasing concentrations of H_2_O_2_ (Figure 1A). *At*AMY3 was inhibited in a dose and time-dependent manner by H_2_O_2_ treatments, although only with high concentration, i.e., 1 mM H_2_O_2,_ as well as long incubation time, i.e., 1 h, the enzyme activity was completely blocked (Figure 1A). 1 h incubation in the presence of 1 mM H_2_O_2_ caused oxidation of *At*AMY3, which could not be reversed by DTT (Figure 1B).

H_2_O_2_ is particularly reactive on cysteine thiols, leading to a sequential oxidation of sulfhydryl groups going through sulfenic (−SOH), sulfinic (−SO_2_H) and sulfonic (−SO_3_H) acid states, the last two of which being irreversible (Supplementary Figure S1A) (Santelia et al., 2015). When cysteines are involved in the catalysis, oxidation affects enzyme activity and irreversible oxidation (i.e., inactivation) can be prevented by thiol modification such as S-glutathionylation. *In vivo* S-glutathionylation mainly occurs by reaction between reduced GSH and the sulfenic state of cysteine (Supplementary Figure S1A). For this reason *At*AMY3 was simultaneously treated with 1 mM H_2_O_2_ plus 5 mM GSH and the enzyme activity was measured before and after DTT reduction. The simultaneous presence of H_2_O_2_ and GSH resulted in a completely reversible inhibition of *At*AMY3 activity (Figure 1B), suggesting that *At*AMY3 could be reversibly modified by glutathione and this may be a mechanism for regulating its activity.

### *At*AMY3 Activity Is Modulated by GSH:GSSG Ratio and Glutaredoxins Speed Up the Reactivation Process

**FIGURE 2:**
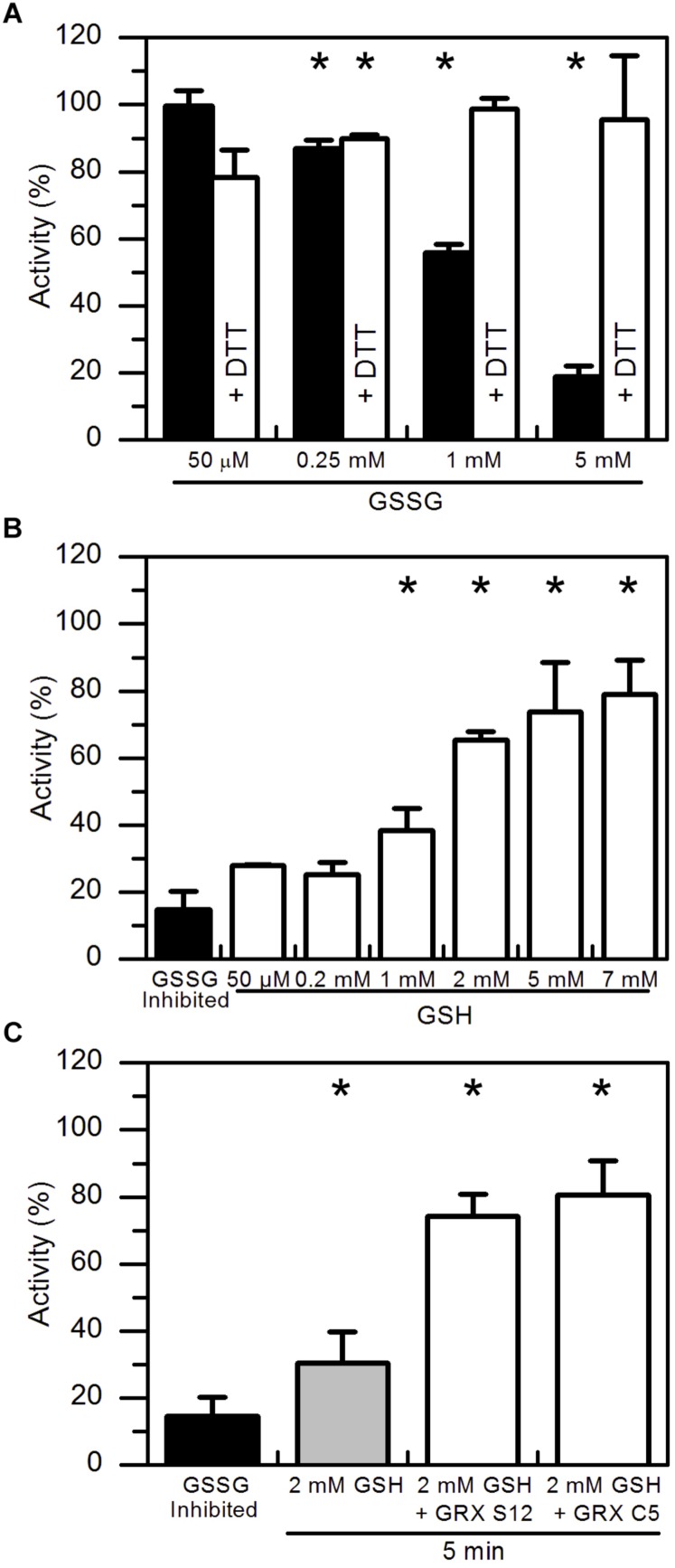
Reactivation of GSSG-inhibited *At*AMY3 by GSH and effect of GRXs on the reactivation rate. **(A)** Inactivation of *At*AMY3 after 1 h incubation with 50 μM, 0.25 mM, 1 mM, and 5 mM GSSG (black bars). The reversibility of the inhibition was tested by incubating samples for additional 30 min in presence of 80 mM DTT (white bars). Activities are expressed as percentages of reduced sample obtained by incubating glutathionylated *At*AMY3 in presence of 80 mM DTT for 30 min at 25°C. Data are reported as mean ± SD (*n* = 3). Significant reduction in enzyme activity compared to the pre-reduced enzyme was determined based on *P*-values obtained from Student’s *t* test. ^*^P < 0.01. **(B)** Recovery of catalytic activity of *At*AMY3 pre-treated with GSSG (black bar) by increasing concentrations of GSH. GSSG-inhibited samples (black bar) were incubated for 30 min at 25°C at different concentrations of GSH (white bars). Activities are expressed as percentages of fully active *At*AMY3, obtained by incubating GSSG-inhibited enzyme for 30 min in presence of 80 mM DTT. Data are reported as mean ± SD (*n* = 3). Significant increase in enzyme activity compared to the GSSG-treated enzyme was determined based on *P*-values obtained from Student’s *t* test. ^*^P < 0.01. **(C)** GSSG-inhibited *At*AMY3 (black bar) was incubated with 2 mM GSH in absence (gray bar) or presence (white bars) of GRX S12 or C5. Activities are expressed as percentages of fully active *At*AMY3, obtained by incubating GSSG-inhibited enzyme with 80 mM DTT for 30 min. Data are reported as mean ± SD (*n* = 3). Significant increase in enzyme activity compared to the GSSG-treated enzyme was determined based on *P*-values obtained from Student’s *t* test. ^*^P < 0.01.

*In vitro* protein glutathionylation can be achieved by the addition of GSSG. GSSG spontaneously reacts faster with the deprotonated rather than protonated sulfhydryl group of cysteine residues (Supplementary Figure S1B). To test the effect of GSSG on the enzyme activity, pre-reduced *At*AMY3 was incubated for 30 min at 25°C at increasing GSSG concentrations. As expected, GSSG treatment inhibited the enzyme activity in a dose-dependent manner (Figure 2A, black bars) and such inhibitions were completely abolished by a second incubation in the presence DTT (Figure 2A, white bars).

In plants, GSH is the main form of glutathione and only small amounts of GSSG are found inside the cell (Foyer and Noctor, 2011). Being the major endogenous antioxidant, GSH is continuously regenerated from GSSG by glutathione reductase (GR) consuming reducing power (Foyer and Noctor, 2011). However, under stress conditions, concentration of both forms of glutathione varies, often leading to a decrease in the GSH:GSSG ratio (Noctor et al., 2012; Hasanuzzaman et al., 2017). Hence, the effect of GSH on GSSG pre-treated *At*AMY3 was tested. As shown in Figure 2B, increasing concentrations of GSH allowed an almost complete restoration of *At*AMY3 activity after 30 min incubation, suggesting that the enzyme can be effectively regulated by the simple change of the GSH concentration (Supplementary Figure S1B).

GRXs are redoxins able to control the thiol-based post-translational modifications of target enzymes, specifically reducing glutathione-mixed disulfides (Meyer et al., 2008). The ability of GRXs to reverse GSSG-mediated inhibition of *At*AMY3 was assayed by measuring α-amylase activity upon 5 min incubation with GRX S12 and GRX C5 plus 2 mM GSH (Figure 2C). Adding these GRXs led to a recovery of about 80% of the maximal activity, a value close to that obtained after 30 min incubation in the presence of 5–7 mM GSH alone (Figure 2B). Taken together, the data indicate that GRXs play a role in the regulation of *At*AMY3 activity speeding up the reactivation of the enzyme (Supplementary Figure S1B).

### Both TRX and GRX Are Required to Completely Restore *At*AMY3 Activity

**FIGURE 3:**
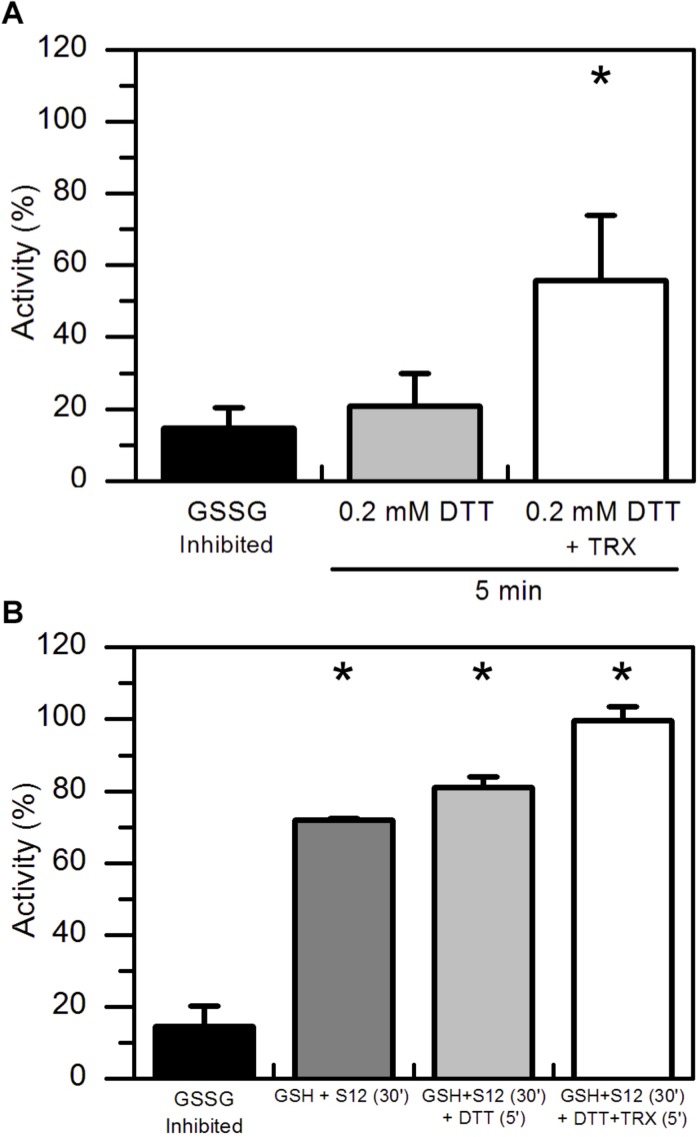
TRX-dependent reactivation of GSSG-inhibited *At*AMY3 and effect of both GRX S12 and TRX. **(A)** GSSG-treated *At*AMY3 (black bar) was incubated in presence of 0.2 mM DTT without (gray bar) or with (white bar) 10 μM TRX. Activities are expressed as percentage of fully reduced samples obtained after 30 min incubation of GSSG-inhibited *At*AMY3 with 80 mM DTT. Data are reported as mean ± SD (*n* = 3). Significant increase in enzyme activity compared to the GSSG-inhibited enzyme was determined based on *P*-values obtained from Student’s *t* test. ^*^P < 0.01. **(B)** GSSG-inhibited *At*AMY3 (black bar) was incubated for 30 min with 2 mM GSH and 5 μM GRX S12 (gray bar). After 30 min incubation, 0.2 mM DTT was added to the mixture either in the absence (pale gray bar) or in the presence (white bar) of commercially available TRX. Incubation was extended for another 5 min at 25°C before measuring *At*AMY3 activity. Activities are expressed as percentage of fully reduced samples obtained after 30 min incubation of GSSG-inhibited *At*MY3 with 80 mM DTT. Data are reported as mean ± SD (*n* = 2). Significant increase in enzyme activity compared to the GSSG-inhibited enzyme was determined based on *P*-values obtained from Student’s *t* test. ^*^P < 0.01.

Considering that GSSG-inhibited *At*AMY3 did not fully restore its activity even in the presence of GRXs (Figure 2C), we speculated that the mixed disulfide bridge occurring between GSSG and the thiol group of one cysteine could be rapidly attacked by the thiol group (−SH) of a second cysteine residue to form a stable disulfide (-SS-) and releasing GSH (Supplementary Figure S1C). To test this hypothesis GSSG-inhibited *At*AMY3 was incubated with 0.2 mM DTT in the presence or absence of *E. coli* TRX. Together with GRXs, TRXs belong to the redoxins family and are well known to efficiently reduce protein disulfides. As shown in Figure 3A, within 5 min incubation TRX partially restores *At*AMY3 activity, corroborating the initial hypothesis of the formation of a disulfide bridge in response to GSSG treatment.

To further test whether GSSG treatment led to the formation of both thiol modifications, sequential measurements of the recovery of *At*AMY3 activity upon a first incubation with GRX S12 followed by a second incubation with TRX were performed. To ascertain that GRX S12-dependent reduction of *At*AMY3 was completed before the addition of TRX, an experiment was performed showing clearly that already after 5 min incubation the reactivation of *At*AMY3 by GRX S12 was completed (Supplementary Figure S2). Similar to previous results (Figure 2C), only 80% of the maximum activity was recovered even after longer incubation (Supplementary Figure S2).

Once confirmed that the reactivation of *At*AMY3 by GRX S12 was completed after 5 min incubation (Supplementary Figure S2), 0.2 mM DTT with or without 10 μM TRX was added and the incubation extended for 5 min. As shown in Figure 3B the activity of *At*AMY3 was completely restored only after incubation with both redoxins (i.e., GRX and TRX). Altogether, these results support the conclusion that GSSG could react with *At*AMY3 inducing the formation of both glutathionylation and disulfide bond.

### At Least Three Different *At*AMY3 Cysteine Residues Are Modified by GSSG

**FIGURE 4:**
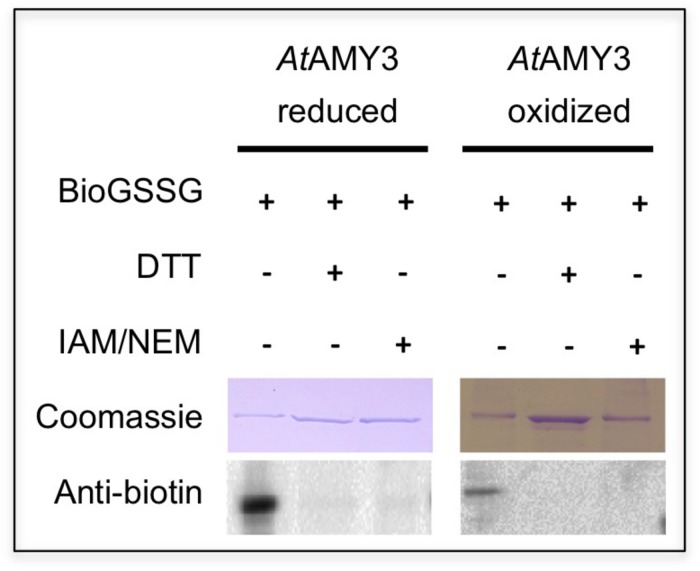
Effect of BioGSSG incubation on reduced and oxidized *At*AMY3. Protein samples (2 μM) were mixed with 2 mM BioGSSG for 1 h prior to separation on a non-reducing 12.5% SDS-PAGE and transfer to a nitrocellulose membrane. The reversibility of the reaction was assessed by a 30 min incubation with 80 mM DTT. As negative control, 2 μM protein sample was alkylated with 100 mM IAM and 20 mM NEM before BioGSSG treatment.

As reported in Seung et al. (2013), *At*AMY3 is regulated by dithiol/disulfide exchange occurring between the cysteine residues Cys499 and Cys587. To analyze the putative involvement of Cys499 and Cys587 in both PTMs (i.e., dithiol/disulfide exchange and glutathionylation), pre-reduced and pre-oxidized *At*AMY3 were incubated in the presence of BioGSSG (Figure 4). This BioGSSG assay detects the formation of a mixed disulfide between the accessible cysteines in the target enzyme and BioGSSG. After the formation of a mixed disulfide, antibiotin antibodies can easily detect biotin. If no reaction occurs between BioGSSG and recombinant enzyme, no signal is obtained.

For comparison, all single cysteine to serine *At*AMY3 mutants were subjected to BioGSSG assay (Supplementary Figure S3). Both pre-reduced and pre-oxidized wild-type *At*AMY3 showed a clear signal that disappeared following incubation with DTT and was prevented by alkylation with IAM and NEM (Figure 4). The same was observed for all single Cys to Ser *At*AMY3 mutants in their oxidized form (Supplementary Figure S3). The positive immunodetection in all samples allowed us to conclude that more than one cysteine residue is subject to S-glutathionylation in *At*AMY3 and that Cys499 and Cys587 are not the only cysteine residues targeted by S-glutathionylation. If that was the case, no signal would have been observed in the pre-oxidized *At*AMY3 sample.

Albeit not all cysteine residues were found exposed and accessible to DTNB (i.e., out of the nine cysteines present in the primary sequence of *At*AMY3 only 7.7 were detected by DTNB) (Table 1), just over three cysteine residues were found modified by GSSG, as calculated by the difference of free cysteine in fully reduced and GSSG-treated *At*AMY3 (Table 1), in agreement with BioGSSG assay.

**TABLE 1:** 5,5′-dithiobis(2-nitrobenzoic acid) (DTNB) analysis of free thiols in *At*AMY3 under reducing or GSSG-treated conditions.

	**No. of free thiols (-SH)**
Reduced *At*AMY3	7.7 ± 1.1
GSSG-treated *At*AMY3	4.2 ± 1.2

*Results are mean ± SD (*n* = 2).*

### Determination of p*K*_a_ Value

Unreactive cysteines are characterized by p*K*_a_ values around 8.5, whereas reactive cysteines are typically surrounded by unusual microenvironment that destabilizes the unreactive protonated form of cysteine residues through electrostatic interactions, significantly decreasing their p*K*_a_ value (Roos et al., 2013). IAM is a strong alkylating agent able to react specifically with thiolate anion (-S^–^). When cysteine residues are involved in the catalytic process, the p*K*_a_ of catalytic relevant cysteines can thus be determined by measuring the pH dependency of IAM inactivation.

**FIGURE 5:**
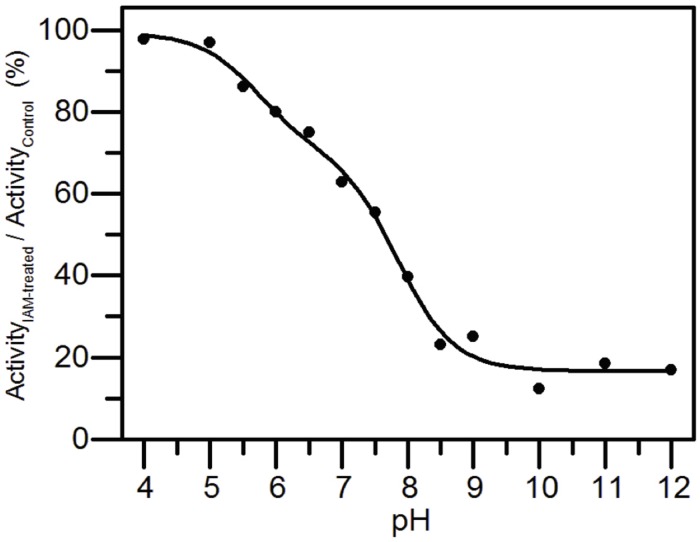
p*K*_a_ determination of the catalytic cysteines of *At*AMY3. *At*AMY3 was incubated in different buffers ranging from pH 4 to 12 in presence or absence of IAM before measuring the activity. The residual activity at each pH value was calculated as percentage of inhibition between IAM-treated and untreated samples, and expressed as a function of pH. The obtained curve was fitted by non-linear regression with two-p*K*_a_ dependence. Results are mean ± SD (*n* = 3; *SD* < 10% are omitted for clarity).

*At*AMY3 was treated with IAM at different pH values and the activities were compared with those measured at the same pH but in absence of the alkylating reagent. Experimental data were fit both to a one-p*K*_a_-dependent (Supplementary Figure S4) and to a two-p*K*_a_-dependent event (Figure 5). However, the best fitting returned two p*K*_a_ values, the first p*K*_a_ was 5.70 ± 0.28, while the second p*K*_a_ was 7.83 ± 0.12.

The finding of a two inflections curve was also in agreement with the low or even absence of activity measured in the single C499S and C587S *At*AMY3 variants (Seung et al., 2013). Moreover, since Cys499 and Cys587 were also responsible for the TRX-dependent regulation, the present finding supports the involvement of at least one and the same cysteine in both redox modifications.

## Discussion

Most organisms constantly produce ROS because of metabolic processes as photosynthesis and respiration. However, unfavorable environmental conditions such as varying temperatures, light intensities and water availability, rapidly modify ROS production. In chloroplasts, several kind of stresses affect photosynthetic efficiency augmenting singlet oxygen (^1^O_2_), superoxide (O_2_^–^), hydroxyl radical (HO) and hydrogen peroxide (H_2_O_2_) production. Among them, the long-lived H_2_O_2_ molecule has received particular attention acting in both signal transduction and regulation of enzyme activity (Bienert et al., 2007; Sharma et al., 2012). Depending on H_2_O_2_ concentration and on the reactivity of cysteine residues, protein thiols can undergo several reversible or irreversible modifications (Supplementary Figure S1). The formation of disulfide and S-glutathionylation are known mechanisms altering enzyme activity (Zaffagnini et al., 2019).

Differently from other α-amylases, *At*AMY3 localizes in the stroma of chloroplast of both guard and mesophyll cells and its activity is strictly regulated by TRX*f*1. Light-driven reduction of the regulatory disulfide bridge between Cys499 and Cys587 activates *At*AMY3 (Seung et al., 2013), presumably leading to a diurnal degradation of transitory starch. Hitherto *At*AMY3 is a unique α-amylase subject to TRX-regulation, a type of regulation in line with the physiological functions ascribed to *At*AMY3, covering stress-induced starch degradation in mesophyll cells and the stimulation of stomatal opening in guard cells (Horrer et al., 2016; Thalmann et al., 2016).

However, the stress response implies an increase in the concentration of H_2_O_2_. The aim of the present study was to elucidate using *in vitro* biochemical approaches whether and how the activity of *At*AMY3 is redox-regulated and the catalytic cysteines protected against overoxidation and reactivated.

Already more than 20 years ago, the inhibitory effect caused by S-glutathionylation of the catalytic Cys95 in barley AMY1 was reported (Søgaard et al., 1993). As for the cytosolic isoforms of Arabidopsis, AMY1 from barley lacks an N-terminal ∼50 kDa domain (Supplementary Figure S5). However, the C-terminal domain of *At*AMY3 and barley AMY1 is highly conserved (46.3% identity and 74.6% similar, based on the comparison between the full-length AMY1 and the last 418 amino acids at the C-terminal of *At*AMY3) suggesting that Cys587 (corresponding to Cys95 of barley AMY1) would be target of glutathionylation. Furthermore, a more recent evidence comes from an *in vivo* study that identified the sulfenylated (–SOH) form of *At*AMY3 in Arabidopsis cells treated with 1 mM H_2_O_2_ (De Smet et al., 2018). This first oxidative state of protein thiols can be followed by further oxidation to sulfinic (−SO_2_H) and sulfonic (−SO_3_H) acids, considered irreversible oxidation (Supplementary Figure S1A). Alternatively, the sulfenylated form can form disulfides reacting with protein or low molecular weight thiols (Supplementary Figure S1A).

**FIGURE 6:**
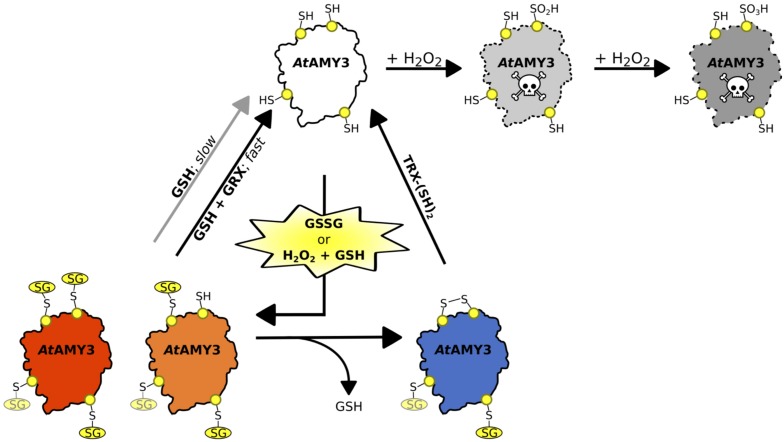
Schematic illustration of the proposed redox-regulatory model of *At*AMY3 activity. As suggested by DTNB assay, 3.5 cys residues were modified by GSSG; three of them are highlighted in yellow while the fourth is shown in light yellow. In the model, *At*AMY3 (white) is fully active when reduced (–SH), but it can be irreversibly inactivated when oxidized to sulfinic (pale gray) or sulfonic (gray) acid forms by H_2_O_2_. However, if oxidation occurs in the presence of H_2_O_2_ and GSH or is mediated by GSSG, *At*AMY3 is efficiently protected from overoxidation via S-glutathionylation. S-glutathionylation can lead to a partially (orange) or fully (red) modified enzyme. In case of partial S-glutathionylation (orange), an intramolecular disulfide (blue) can be formed releasing GSH. Thioredoxin (TRX) can activate *At*AMY3 by reducing this disulfide (white). Similarly, fully glutathionylated *At*AMY3 (red) can be efficiently activated by GSH through a slow (gray arrow) or a fast (black arrow) process depending on absence or presence of class I glutaredoxin (GRX), respectively.

For this reason, the effect of H_2_O_2_, GSH plus H_2_O_2_ and GSSG were tested on *At*AMY3 activity, demonstrating that *At*AMY3 can be irreversibly oxidized by H_2_O_2_ and reversibly glutathionylated by GSH plus H_2_O_2_ or GSSG (Figures 1B, 2A, 6). Thus, the sulfenylated form of *At*AMY3 is target of S-glutathionylation *in vitro*, and this PTM likely protects the enzyme from irreversible oxidation.

At least three cysteine residues were modified by a GSSG treatment (Table 1), two of which, judging from the results obtained by the p*K*_a_ analysis (Figure 5), are involved in the catalytic activity. Indeed, this type of analysis returns the p*K*_a_ value only of cysteines involved in the enzymatic activity, whereas it cannot detect cysteine residues that do not affect enzyme activity even if they are modified. In Seung et al. (2013), single Cys to Ser mutants of residues 499 and 587 were ∼90% less active than wild-type *At*AMY3, suggesting that two out of the three GSSG-modified cysteine residues could be Cys499 and Cys587.

The same pair of cysteine residues is also responsible for the TRX-dependent regulation of *At*AMY3 (Seung et al., 2013). Taking this into consideration and the fact that both redoxins (i.e., GRX and TRX) were required for a fast (Figures 2C, 3A) and complete (Figure 3B) reactivation of *At*AMY3 activity, the proposed model suggests that during oxidative treatments leading to glutathionylation, partially modified enzyme formed a homodisulfide releasing GSH (Figure 6).

The slow and non-protein-assisted reactivation of *At*AMY3 by GSH (Figure 2B) adds complexity to the system. Although varying across tissues and subcellular compartments, in the absence of stress the GSH:GSSG ratio is typically considered very high (Schwarzländer et al., 2016). This could suggest that on the way to recover from stress-induced ROS production, the activity of *At*AMY3 would be fine-modulated by the high concentration of GSH. Considering the role of *At*AMY3 in stress response (Thalmann et al., 2016), it seems reasonable to assume that the biochemical features of the enzyme equipped *At*AMY3 to overcome the burst of oxidative stress. Equally true, however, is that a rapid reactivation of the enzymatic activity is required to the onset of plant response to stress.

In the tangled scenario of the redox regulation, *At*AMY3 offers a good example of the cross-talk between redox-dependent protein modifications (i.e., dithiol/disulfide exchange and S-glutathionylation) in the fine-tuning of enzymatic activity. Future research will be required to validate the role of these PTMs *in vivo*.

## Data Availability

The raw data supporting the conclusions of this manuscript will be made available by the authors, without undue reservation, to any qualified researcher.

## Author Contributions

CP, LG, LD, and NR purified the recombinant proteins. CP and LG performed the BioGSSG assays and biochemical characterization. FS and DS coordinated the experiments, supervised the whole research, and wrote the manuscript. All authors discussed the data and reviewed the manuscript.

## Conflict of Interest Statement

The authors declare that the research was conducted in the absence of any commercial or financial relationships that could be construed as a potential conflict of interest.

## Footnotes

1 http://espript.ibcp.fr
