# Supplementary material for: The Thioredoxin-Regulated α-Amylase 3 of Arabidopsis thaliana Is a Target of S-Glutathionylation
Source: Front Plant Sci. 2019 Jul 31;10:993. doi: 10.3389/fpls.2019.00993 (PMC6685290; doi:10.3389/fpls.2019.00993)
Supplement: Supplementary file 1 [file Data_Sheet_1.PDF]

Figure S1

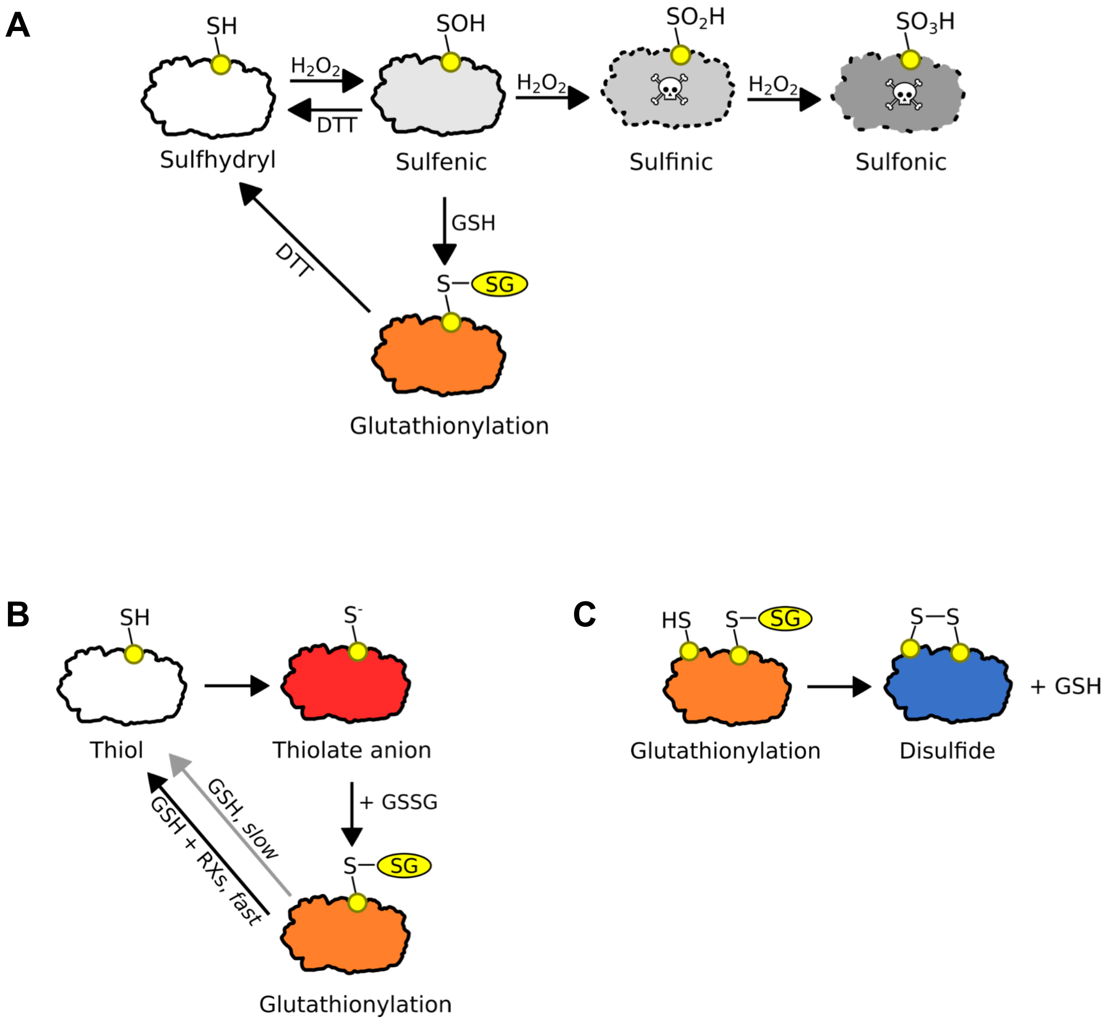

Figure S1 | Schematic overview of the major oxidative thiol modifications that can occur in response to oxidative stress. **(A)** Cysteine thiols (-SH; white) are easily oxidized by hydrogen peroxide (H_2O_2) leading to a sequential oxidation going through sulfenic (-SOH; pile grey), sulfinic (-SO_2H; grey) and sulfonic (-SO_3H; dark grey) acid states, the last two of which caused irreversible damage. However, sulfenic acid (white) can also form a mixed disulfide with GSH leading to protein glutathionylation (orange). *In vitro* this modification can be removed by DTT restoring the active form of the enzyme. **(B)** At physiological pH reactive cysteines (white) are represented by their thiolate anion (red), then easily attacked by GSSG leading to protein S-glutathionylation (orange). A slow and non-redoxins (RXs) assisted (grey arrow) and a fast and RXs assisted (black arrow) mechanisms of protein deglutathionylation can remove the PTM restoring the enzymatic activity. **(C)** S-glutathionylated cysteine (orange) can be attacked by a second thiol group (-SH) forming a stable disulfide (-SS-; blue) and releasing GSH.

Figure S2

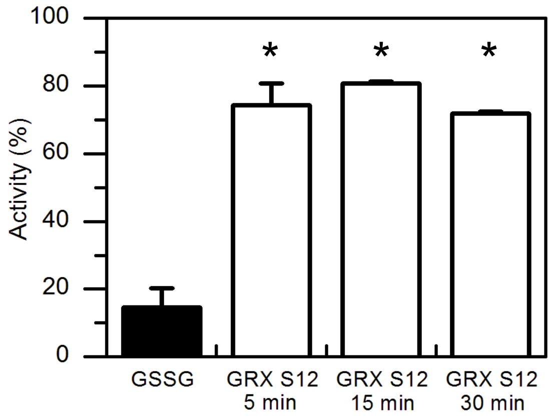

Figure S2 | Time-dependent reactivation of GSSG-inhibited *At*AMY3 by GRX S12. GSSG-inhibited *At*AMY3 (black bar) was 5, 15 and 30 min incubated with 2 mM GSH and 5 µM GRX S12 (white bar). Activities are expressed as percentage of fully reduced samples obtained after 30 minutes incubation of GSSG-inhibited *At*MY3 with 80 mM DTT. Data are reported as mean ± SD (n = 3). Comparisons were made using p-value obtained from Student's *t*-test performed on GSSG-treated sample. *, $p<0.01$.

Figure S3

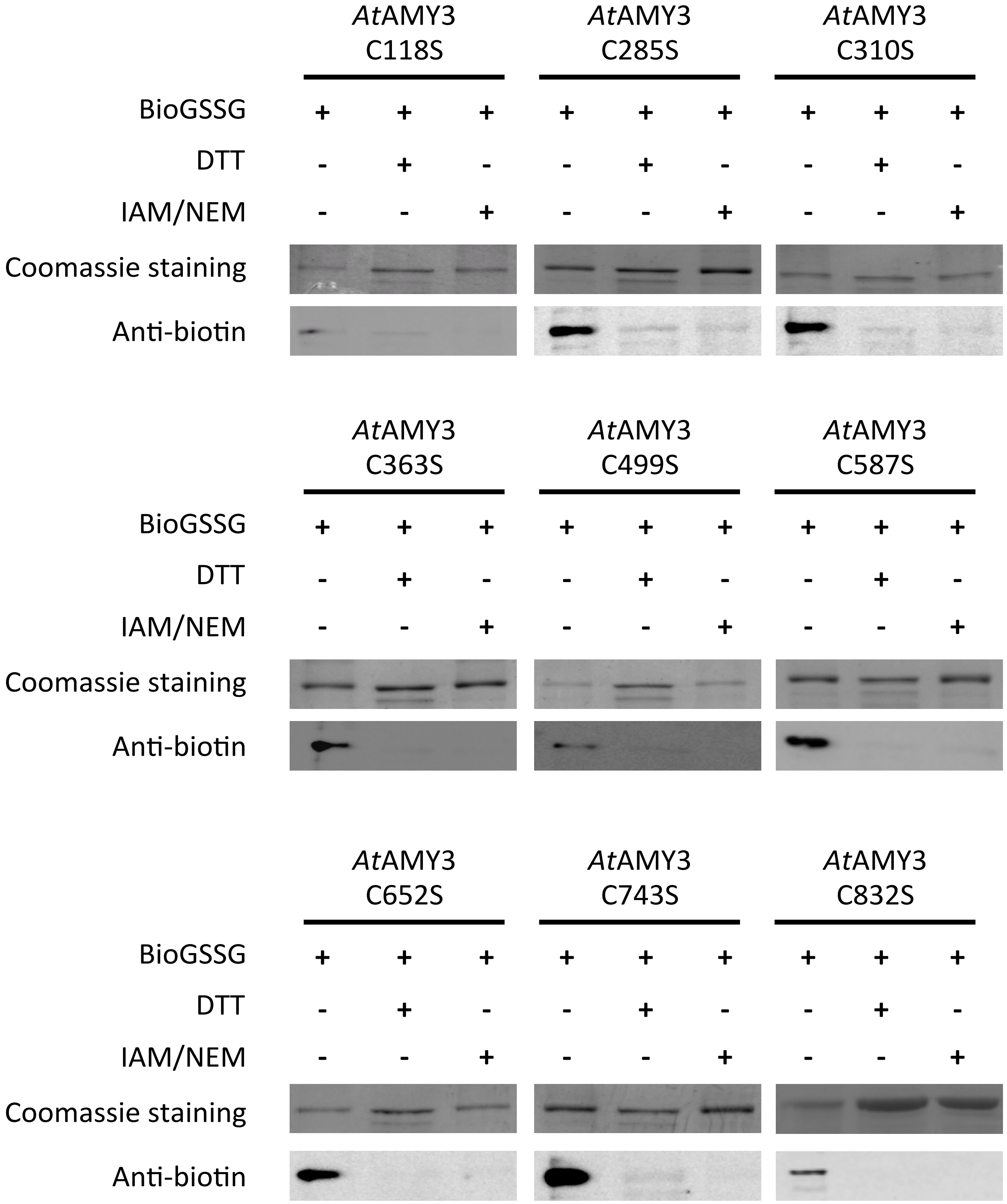

Figure S3 | Effect of BioGSSG incubation on single Cys to Ser mutants of pre-oxidized *At*AMY3. Protein samples (2 µM) were mixed with 2 mM BioGSSG for 1 h prior to separation on a non-reducing 12.5% SDS-PAGE and transfer to a nitrocellulose membrane. The reversibility of the reaction was assessed by a 30 min incubation with 80 mM DTT. As negative control, 2 µM protein sample was alkylated with 100 mM IAM and 20 mM NEM before BioGSSG treatment.

Figure S4

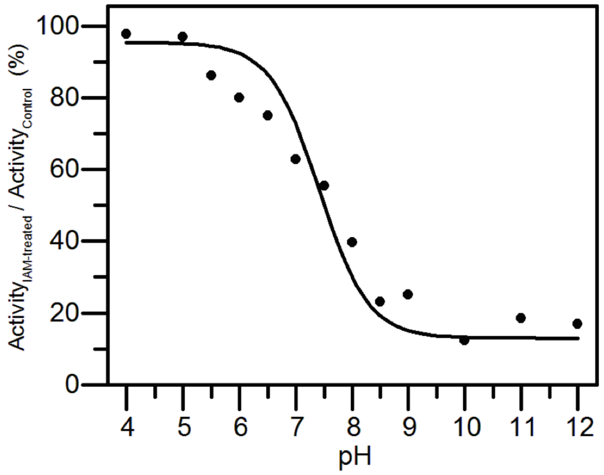

Figure S4 | pK_a determination of the catalytic cysteines of *At*AMY3. *At*AMY3 was incubated in different buffers ranging from pH 4 to 12 in presence or absence of IAM before measuring the activity. The residual activity at each pH value was calculated as percentage of inhibition between IAM-treated and untreated samples, and expressed as a function of pH. The obtained curve was fitted by non-linear regression with one-pK_a dependence. Results are mean ± SD (n = 3; SD < 10% are omitted for clarity).

Figure S5

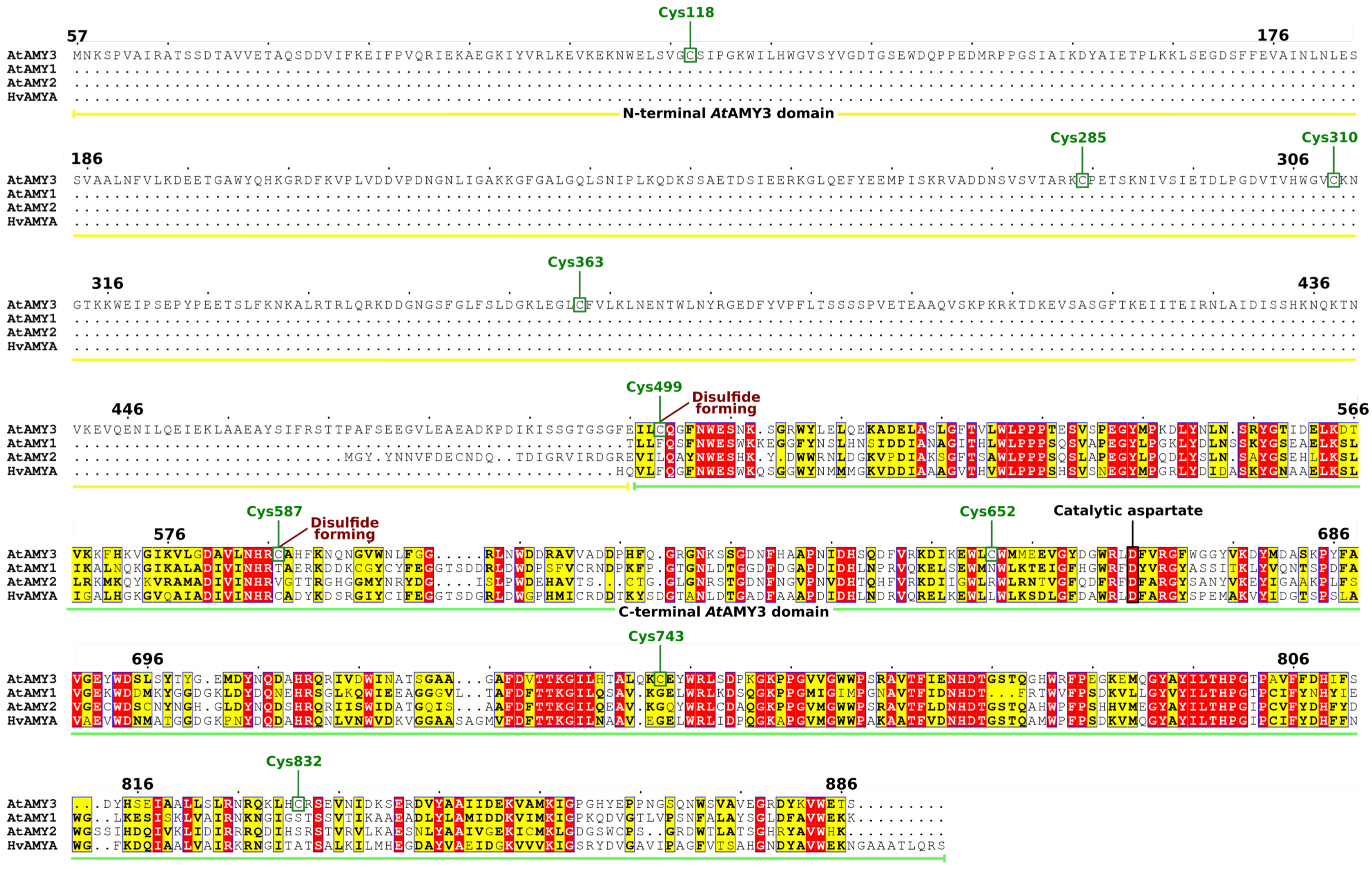

Figure S5 | Sequence alignment of the three α-amylases (*At*AMY1-3) encode by the Arabidopsis genome and of *Hordeum vulgare* α-amylase A (*Hv*AMYA). The alignment was performed with Clustal Omega. The N-terminal domain, characteristic of *At*AMY3, is highlighted with a yellow line. In the conserved C-terminal domain (green line), Cys residues involved in the thioredoxin-dependent regulation are reported. Identical residues are written with white characters and boxed in red; similar residues are written with black bold characters and boxed in yellow.
